# Hospital Administration

**Published:** 1898-09-24

**Authors:** 


### HOSPITAL ADMINISTRATION.

### A CONTINENTAL HOSPITAL.

(By a Correspondent.)

I am sitting outside the Palace at Versailles with the sun streaming down in unclouded brilliance upon the white stone buildings and baked gravel yards and stiff Italian gardens and statuary, and I am tempted as I sit in the shade of the chestnuts to jot down a few impressions of the Hospital of Geneva that I visited yesterday.

When a doctor visits a hospital he is inclined to look at it solely from a medical point of view; but, having in my mind at the time a special interest in the problems of nursing, I made inquiries which, I think, may not be without interest to the managers of English hospitals. I called at the hospital at half-past nine in the morning, so that I might be present at the operations, which are done in the early hours, and was at once shown to the operating theatre and was there welcomed by the surgeons, who were in the middle of th first operation of the day. When this was over I explained that I was not only anxious to see over the hospital myself, but that I wished also to take over it three lady friends (nurses) who were with me—one the matron of a provincial hospital, one a sister of a large London hospital, and the third a nurse in a small London hospital. Permission was most courteously given; so that after spending about two hours in the operating theatre I joined the nurses, and we went over the hospital together.

In comparing the theatre nursing with that of one of our English hospitals, I think that the points that struck me most were the want of that special organisation and machine-like adaptation of each one to her duties to which one is so accustomed at home. Instead of a theatre sister and ward sister and a nurse and a probationer there were present two nurses, who seemed of equal authority, and who did indiscriminately what each happened to be asked to do. There was none of that system of bowls of lotion and sponges being continually brought to the operator, and the soiled lotion and sponges being as systematically carried away by the nurse in charge of it. When sponges were wanted the surgeons had to ask for them, and the nurse brought a few more in her hand; and when they were used they accumulated in a heap on the edge of the operating-table. One of the nurses, too, cut the ligatures, although three surgeons were assisting in the operation, and handed them with her hands and not with forceps. I was interested, too, to notice that, although there was every facility for the most perfect sterilisation, yet it did not appear that the instruments that were being used for the operation had been specially prepared immediately preceding the operation. At any rate, between two operations I did not see the instruments from the first one cleared away and fresh ones taken direct from the boiling water brought in. Once when some retractors were wanted in the middle of an operation for removing malignant cervical glands the nurses got them from the outside cupboard, brought them in her hands, with a considerable amount of handling, and after thorough washing in antiseptic lotion, were used without being boiled. There was no theatre beadle about, so that I suppose the nurses had full charge of the sterilisation of the instruments. The want of that organised supervision by a sister which is so striking a feature of our own hospitals again forced itself upon me in a small but most important trifle. I had been talking to one of the surgeons, at the close of a somewhat protracted operation in the lumbar region, of the greater amount of covering that would have been applied to the limbs in a similar operation in England, and the much less exposure of the lower part of the body and limbs that would have taken place, owing to the special stress which is laid upon retaining the heat of the body and the avoidance of any preventable loss of it during long operations. "Do you," I asked, "however, have a hot-water bath in your operating-table?" "Oh yes," he answered, and, as I stepped forward to feel the heat, added, "I suppose it has been filled, nurse?" It had not!

We were conducted over the wards, and everywhere met with the greatest kindness. No trouble was too great and no amount of time too much to spend in showing us everything we wished to see and in answering every question which our somewhat limited knowledge of French enabled us to put. Whatever faults there may be in the hospital administration, certainly churlishness is not one, for doctors, nurses, and porters alike showed an example of courtliness and kindness to strangers which the staff of many English hospitals would do well to imitate. We were anxious to know some details of the nursing training, and management, so as to compare it with our own system. We found that there are two methods which at present are working side by side at this hospital. The first, and the older one, is a scheme of farming out nurses. There is at Berne a great central institution, worked, as far as I could gather, on family lines, with a "father" and a "mother" of the home. The nurses are called "deaconesses," and have a year's training in surgical, medical, and midwifery work, pass an examination at the home, and are then sent out to staff the majority of the Swiss hospitals. There is no "matron" of the hospital. The chief administrative duties which in England are done by the matron are here done by an important functionary entitled the "director," who seemed to combine to a large extent the functions of matron, house governor, and secretary. The nurses in each block were under the control of the sister of the block, so that considerable diversity of administration seemed possible where there was no matron going round to correct, advise, and order. When a nurse is ill she is sent back again to the central institution whenever possible, and only when suddenly and seriously ill is she warded. For each nurse a sum of about £18 a year is paid by the hospital to the central nursing home, but when I further asked how much of this was paid back by the home to the nurse my informant laughed amusedly and shrugged his shoulders. I explained to him that in England ladies of title and position took up nursing, and that, broadly-speaking, nurses were of good social position. "Here," he replied, "it is not so; nurses are drawn from a by no means high class." "But don't you," I queried, "in a splendid hospital like this, with its six or seven hundred beds, train your own nurses?" "That," he replied, "is the second method that I spoke to you of. We have during the last year or two tried to work up a training-school, but so far I can say but little of it. It is not successful; we have but two or three probationers. The people of Geneva are not suited to become nurses, their heads are too full of other things." I may here add that at the present time I am in communication with several hospitals on the Continent with a view to discovering whether English women would be admitted as probationers, and if so on what terms, for there might be an important opening in this direction for women who wanted to travel, to learn French, and to be trained in Continental methods of nursing at the same time. Just as many a doctor is attracted to do an extra year at Brussels, or Berlin, or Paris, so our best nurses may yet find it a valuable addition to their knowledge to put in a year at a first-rate Continental hospital—but of this more later.

I should omit an important feature of this hospital which struck us all if I did not refer to the "pavillons." During the summer a large number of wards are closed and handed over to the painter, and allowed to rest and purify for nearly six months, and the patients are transferred to open wooden sheds erected on piles about 3 ft. from the ground. Only the roof and floor are permanently covered; the sides and ends are quite open, excepting for canvas screens, which are drawn on rings and poles all round. Those on the windward side are drawn, the others are all drawn back so that practically the patients are night and day in the open air. After operations of the most serious character the patients are at once taken out here, so that it must not be looked upon as a convalescent branch, but these "pavillons" are really outdoor hospital wards. It was charming to see a sparrow hop in, pick a bit of bread that a patient had laid on her coverlet, and fly out again. The beds were all full, and the patients most happy and comfortable. There were about twenty beds in each "pavillon." I was specially interested in this because last summer I tried a somewhat similar experiment for medical cases at my hospital at Loughton, which adjoins the Epping Forest, but I found the English fear of "catching cold" and "night air" a constant source of trouble. I hope, however, to recommence experiments ere long upon a more carefully guarded scale, as the experience here was decidedly favourable, and the advantage of giving the hospital wards a few months' rest every year is an object of no mean or slight value. I have much more to add, but this may suffice to give a little idea of a hospital abroad.

